# Should Care Robots Answer? Rethinking Moral Answerability Beyond Humans

**DOI:** 10.1111/nin.70150

**Published:** 2026-08-02

**Authors:** Mario Kropf

**Affiliations:** ^1^ Department for Ethics and Social Studies, Department of Moral Theology, Faculty of Catholic Theology University of Graz Graz Austria

**Keywords:** answerability, care robots, human–robot interaction, moral responsibility, reactive attitudes, social robotics

## Abstract

AI‐based systems increasingly operate in socially and ethically sensitive domains such as healthcare, raising pressing questions about responsibility. This article focuses on a specific form of moral responsibility—answerability—in the context of AI‐based care robots. Although such systems are not moral agents in a human‐like sense, their actions are embedded in social practices in which demands for explanation play a significant role. The article develops a structured analytical framework that conceptualizes answerability as comprising both a subjective dimension (the actor's reasons and justifications) and an objective dimension (the comprehensibility and moral quality of explanations). It thereby clarifies the conditions under which demands for explanation arise and how they can be normatively assessed. Applying this framework to care robots, the article argues that these systems can be meaningfully treated as answerable, even though they are not accountable—in human terms. Concrete care scenarios illustrate that affected individuals may be entitled to demand explanations from robots, despite their technically mediated and limited responses. While current systems can only approximate the requirement of sincerity in a minimal sense, future developments may enable more sophisticated forms of technical answerability without necessarily grounding full moral responsibility.

## 1 Introduction

Moral responsibility is an indispensable component of social and moral practice. People take responsibility for bad outcomes that have occurred, assume obligations based on a role, justify their actions, or bear forward‐looking responsibility. Numerous differentiations and specifications have taken place within the philosophical debate on (moral) responsibility, and AI‐based systems are increasingly being incorporated into the debate. This article deals with a form of moral responsibility regarding AI‐based care robots—namely, answerability. The following considerations refer to Angela M. Smith and other contemporary perspectives but also deviate from them. This is because the relevance of answerability is considered in itself and then specifically in the context of robots. *First*, answerability is presented as an analytical framework and distinguished from attributability and accountability. The *second* step clarifies the extent to which answerability can be understood subjectively and objectively. *Third*, the focus shifts to AI‐based care robots and shows the extent to which answerability can be applied to these machines and what limitations currently still exist. In doing so, the article makes three contributions. (1) It develops a structured framework of answerability (P1–P7) that clarifies its internal components and their interrelations. (2) It specifies the moral quality requirement (P6) in terms of sincerity and orientation toward moral standards, thereby addressing ambiguities in existing accounts. (3) It shows how this framework can be meaningfully applied to AI‐based care robots, while identifying principled limits—most notably regarding sincerity. Although simplified forms of answer can be implemented, the requirement of sincerity—central to the moral quality of explanations—cannot currently be met.

## 2 Answerability as an Analytical Framework

There are numerous proposals regarding (moral) responsibility in general: Hart, for example, distinguishes between four types (role, causal, liability, and capacity) of responsibility (Hart 2008), whereas Vincent discusses two further types (outcome, virtue) (Vincent 2011). A distinction is made, for example, between forward‐looking and backward‐looking responsibility (Van de Poel 2011; Zimmerman 2022), individual (Kaiserman 2021; Kiener 2025) and collective responsibility (Conradie 2023; Taylor 2024), or attributability, answerability, and accountability (Shoemaker 2011; Watson 1996). Smith's considerations serve as a starting point: “[…] to say that an agent is morally responsible for something is to say that that agent is an appropriate target, in principle, of requests for justification regarding that thing […]” (Smith 2015, 103). She refers to reactive attitudes and discusses answerability. These reactive attitudes can be understood as follows: “[…] someone is a morally responsible *agent* insofar as he is an appropriate candidate for at least some of the reactive attitudes on the basis of at least some of his behavior (or perhaps his character)” (Fischer and Ravizza 1998, 6). They refer to Strawson and the “reactive attitudes” (Strawson 1962) that have been discussed extensively since he first introduced the concept. Among other things, these attitudes explain why one expects others to take responsibility and why one takes responsibility oneself^1^—regardless of norms or conditions. In contrast to answerability, attributability means that an action can be attributed to a person because the action expresses their character, beliefs, or even their “deep self” (Mason 2015, 3047). Accountability would entail moral responsibility (thick^2^ blame) for the person in question because the explanation (answerability) is insufficient and others have not been considered sufficiently (Watson 2018, 991).

It is generally assumed that moral responsibility (accountability) requires a moral actor, which I also accept at this point. Appropriate addressees therefore require certain capacities, and specific conditions^3^ must be met. If a nurse (N1) injures a patient (A1) during a routine examination (o1), it is to be expected that the injured person will react with sadness, anger, or fear—as opposed to complete indifference. On the one hand, this reaction suggests that N1 should be held accountable, for example, by demanding an apology. On the other hand, it can be assumed that this reaction will also contribute to N1 taking responsibility and avoiding future misconduct in this regard or acting more carefully. Reactive attitudes can thus be seen as reasons for moral responsibility. And the ability to act on the basis of certain reasons is a prerequisite for moral responsibility (Jeppsson 2016, 684; Nelkin 2016, 371). With regard to answerability, however, it can already be assumed that certain reasons are subjective, which means that it must be clarified what an objective claim might look like. For the following considerations, answerability is developed as an analytical framework that, broadly in line with Smith, explicates the conditions under which actors are treated as answerable in moral practice (Smith 2012, 585). The following premises are introduced sequentially and build upon one another. Together, they identify the conditions under which answerability becomes morally relevant and how demands for explanation relate to subsequent assessments of accountability.

P1^4^: An actor (A) is treated as *answerable* insofar as it is justified to demand an explanation for an action^5^ (x) and its outcome (o).

P2: It is justified to demand an explanation if x or o affects the interests of other actors.

P3: It is also justified to demand an explanation if there are generally good reasons for doing so.

P4: The requested explanation from A refers to subjective reasons for x (or not x).

P5: However, A's subjective explanation does not correspond to an objective justification of why A actually contributed (causally) to o with x.

P6: The degree of moral responsibility (accountability—thick praise and blame) of A depends on the moral quality of this subjective explanation.

P7: A's subjective explanation may lead one to attribute no moral responsibility (accountability—thick praise and blame) to A for x or o.

C: This framework suggests that A is morally *answerable* for x (or not x) when an explanation from A can be justifiably demanded. The degree of moral responsibility (accountability) that may follow is measured by the quality of the subjective explanation. Such an explanation, based on reasons that determine the action, may lead to A being exonerated.

The premises above should not be understood as a deductive syllogism from which a conclusion follows logically. Rather, they serve as an analytical framework that clarifies the conditions under which answerability becomes morally relevant. The aim is not to provide a reductive definition of answerability, but to explicate how justificatory practices, affected interests, and evaluative standards jointly structure its application. The following sections return to these premises selectively in order to examine which aspects of answerability can plausibly be extended to AI‐based care robots and where important limitations remain. Premises P2–P7 do not therefore serve as independent grounds for answerability. Instead, they describe various normative dimensions that structure when and how demands for explanation arise and how their moral quality is evaluated. At first glance, this formulation may appear circular, insofar as the justified demand for an explanation seems to function both as a condition (P1) for and as an indicator of answerability (C). However, this reflects a structural feature of moral practice rather than a conceptual flaw. Practices of demanding and giving reasons are partly constitutive of answerability itself (Caruso and Morris 2017, 844): to treat someone as answerable is, in part, to regard them as an appropriate addressee of justificatory demands. The framework therefore does not seek to ground answerability in independently specifiable criteria, but to make explicit the normative dimensions that are already implicit in such practices.

### 2.1 The Subjectivity of Answerability

Answerability has both a subjective and an objective dimension. This section focuses on its subjective aspect: answerability concerns the reasons actors can give for their actions, rather than causal explanations of outcomes. This means that Anna's explanation will differ from Bernd's, for example, because different actors may act for different reasons. Or, to put it another way: “[…] we are regarding her as ‘answerable for’ the thing in question in virtue of the fact that it reflects her assessment of reasons […]” (Smith 2015, 113). What matters, therefore, is not primarily how an outcome came about, but how the actor understands and justifies their action. This focus on reasons highlights that answerability is tied to the acting subject and their assessment of reasons (P4). At the same time, the causal relationship between A, x, and o does not by itself clarify whether and, if so, to what extent Anna is morally responsible (P5). Rather, moral evaluation depends on how an actor can justify their actions (P6). Otherwise, the subject‐related explanation would lose its central relevance. This becomes clearer in a care context.

Consider a case in which a nurse (Nate) and a patient (Carry) both contribute, through their actions, to another patient (Tom) suffering harm (o2). It seems appropriate for Tom to demand an explanation from Nate and Carry because his interests were directly affected. However, it seems implausible to understand answerability in such a way that Tom asks Nate, for example, why he was injured by Carry, or vice versa, demands an explanation from Carry as to why Nate's actions led to his heart attack. It is about the answer of a specific actor: “[…] the things for which one is answerable reveal one's answers to certain questions, and so can reveal one's mind […]” (Hieronymi 2008, 363). Moreover, the demand for explanation is shaped by relational contexts (Tiffany 2024, 631). This does not imply that only those who personally know Nate or Carry may demand a corresponding answer, but rather that some form of relational connection is required between the actor giving the explanation and the one demanding it. Tom has a stronger claim to an explanation than uninvolved parties, because his interests are directly affected. Relationships are characterized by listening to each other, attributing and taking responsibility, showing reactions, or, in short, being answerable to each other (Hieronymi 2008, 363; Jeppsson 2016, 689; Smith 2015, 120): “Every time we challenge another to defend an opinion, an emotion, or an action, we show that we take her to be answerable for it in more than an ‘attributability’ sense” (Smith 2015, 113). However, answerability is not limited to such cases. In care settings, maintaining trust and mutual understanding can also justify demands for explanation (P3) beyond directly affected individuals. Associated interactions therefore imply moral standards that must be respected by all actors under certain conditions, such as avoiding harm, considering others, helping in emergency situations,^6^ or even sincerity.

Nate and Carry are in a care facility where not only Tom's interests must be considered, but other people live there as well. This community is built on relationships and mutually considering behavior. In order to maintain these interpersonal relationships, it seems essential to have a certain degree of trust in other persons, for example, to anticipate how others will act and why they do so. The relevance of experience and exchange for building and maintaining trust is emphasized by several works (Baier 1986; Luhmann 1989; Nguyen 2022). These considerations suggest that in such cases (e.g., o2), there are good reasons for other residents and nursing staff to demand an explanation from Nate or Carry, even if they are not directly affected. Being directly affected strengthens such claims but is not a necessary condition. Moreover, Nate has certain (role‐based) obligations that go beyond the previous remarks on general moral conduct and require him to provide an appropriate response for his own behavior. Related reasons include at least those just described, and arise from general responsibility practices (Smith 2012, 587–588). The *reactive attitudes* mentioned above make it clear that people react to each other and the associated actions, which expresses such attitudes and refers to the consideration or violation of moral standards.

### 2.2 The Objectivity of Answerability

Answerability is also objective, because the subjective explanation must be comprehensible and meet certain standards. This establishes a connection between subjective reasons (P4), the insufficient causal relationship between A, x, and o (P5), and the moral quality (P6) of an explanation. When Carry justifies her actions by saying that hurting others is fun, a subjective reason for her actions comes to light, but moral standards are disregarded (Singer 1993, 92–93). P4 is fulfilled, P5 is not undermined by this statement, but P6 is not satisfied. The failure to consider the interests of others—because Carry enjoys harming others—reflects an insufficient moral quality of the explanation. Jeppsson puts it this way: “[…] an agent must be responsive to reasons (and perhaps not just reasons in general, but *moral* reasons too) in order to be answerable for what she does” (Jeppsson 2016, 684). However, this still leaves open what P6 requires and how it relates to P4. Moral standards depend on factors such as roles (e.g., by Nate), social conventions, or interpersonal relationships, and need not sharply diverge from subjective standards. The moral quality of an explanation (P6) can be understood in terms of two interrelated requirements: (1) comprehensibility, understood as sincerity and truthfulness, and (2) orientation toward moral standards that are at least broadly acceptable. Let us elaborate on this.

(1) Comprehensibility means that the person acting is sincere, honest, and accordingly tells the truth about why they acted in one way or another. In the previous example, it is conceivable that Nate expresses his own regret about the incident and at the same time is happy that o2 occurred. Even if answering the associated question—whether Carry or Nate is telling the truth—is primarily an empirical task, this requirement of sincerity, truthfulness, and honesty seems to be necessarily linked to the moral quality of the explanation (P6). Singer sums it up by referring to relationships: “[…] it is necessary to take at least some ethical standards seriously, and to be open and honest in living by them – for a life of deception and dishonesty is a furtive life, in which the possibility of discovery always clouds the horizon” (Singer 1993, 327). This is not merely a matter of hoping that Nate and Carry are sincere. Within practices of answerability, there is a justified expectation that explanations be truthful when P2 or P3 apply. A justified demand for explanation entails at least a minimal obligation to provide sincere accounts. This expectation arises from social practices of responsibility, in which agents are interdependent and rely on each other's explanations. Conversely, the interests of Tom or other good reasons speak in favor of Nate and Carry being sincere and giving an explanation that corresponds to the truth. This claim arises from the practice of responsibility, because people act in a social structure, should respect and trust each other, perform and experience certain actions, and thus react to each other (Smith 2012, 588–589). In short, people react to their own behavior and that of others, which makes them interdependent and reliant on each other (Jeppsson 2016, 690). This interdependence is a plausible indication of why answerability should not only be understood as a claim to demand an explanation, but also as a moral obligation to provide truthful information, as in the case of Nate and Carry. With Aristotle, one can say that virtues (such as sincerity) “[…] are within our power and proceed from our will and are as right reasoning dictates” (Aristoteles 2019, 71 NE 1114b).

It cannot be ruled out that subjective explanations may deviate from what is generally considered morally appropriate. This is particularly true if P4 is understood as subjective (internal) and P6 as objective (external). But must it be understood this way? In a similar context, Zimmerman draws attention to the potential dilemma regarding subjective and objective standards: “Suppose that, in addition to there having been no reason to think that taking the pill would be harmful to her fetus, there was very good reason for her to think that *not* taking it *would* be harmful. In this case, Sally's evidence would have been diametrically opposed to the facts. The objective view implies […] that it would have been wrong (*ceteris paribus*) for Sally to take the pill; the subjective view implies that it would have been wrong for her *not* to take it” (Zimmerman 2022, 37). In short, Zimmerman doubts this moral dilemma, and I consider it similarly unproblematic for the considerations on answerability, and more specifically, regarding P4 and P6. But why? Let us turn to the moral standards mentioned.

(2) The reference to moral standards means that such guidelines play a role for Nate or Carry, but not that they must be fulfilled. In Zimmerman's example, Sally acted against an objective standard, but at the same time pursued a subjective standard. In this context, and also considering Nate, it is important that the subjective standard pursued can be described as objectively comprehensible and acceptable—it could, so to speak, become an objective standard. The generalizability of personal moral guidelines required by Kant's categorical imperative^7^ serves as a point of reference here, even if it is not necessary to adhere too closely to his requirement. If a subjective standard that expresses reasons for actions serves exclusively one's own well‐being without even remotely considering the interests of others, it cannot be an objective moral standard. Moral considerations are directed at a broader audience in order to go beyond the individual (Singer 1993, 325–326; Slote 1992, 64). The moral quality of the explanation described in P6 can be understood in a similar way. Sally or Nate's reasoning may not meet the moral standard that other people would have followed, but it seems plausible to claim that it is a moral standard that can be appreciated and described as acceptable—the same cannot be said of Carry. If answerability is understood as moral answerability, it is associated with values such as respect (Vallor et al. 2025, 11–12), avoidance of harm (Beauchamp and Childress 2019, 159), taking responsibility (Strawson 1962, 208), and the maintenance of relationships (Smith 2015, 119–120). Carry's willing and deliberate harming of others expresses a subjective standard, but not reasons that would be reflected in an objective or even objectifiable moral standard (P6)—at least none that could reasonably be described as such. Nate, however, equally violates a moral standard of due care or nonmaleficence, but could justify himself as follows: “It was not my intention to hurt Tom, and I apologize for that.” Sally could react similarly: “I only wanted the best for my child and was convinced that this would prevent her from being harmed.” The moral standards violated here do not contradict the fact that the avoidance of harm or due care fundamentally determines Nate and Sally's actions, nor that the explanations given by both of them have sufficient (positive) moral quality. In short, P6 implies that justifying persons (1) communicate *sincerely* and according to (subjective) truth, and (2) at the same time remain true to *their own* moral standards, which imply a certain degree of objective comprehensibility and acceptability.

The moral quality of the subjective explanation required in P6 refers to P4 and is therefore related to the subject. But in what way are (1) and (2) insufficient to constitute the moral quality in P6? Comprehensibility is a necessary but not sufficient condition for achieving the moral quality described in P6. Otherwise, one would have to claim that Carry fulfills the necessary moral quality by explaining that she likes to hurt other people, assuming that this explanation is comprehensible. Something similar can be seen when Nate, in the previous example, responds to the question of why he acted in this way with the following: “I have no idea” or “I have no idea how that could have happened, I didn't mean to do that and I'm sorry.” In both cases, comprehensibility does not concern the reconstruction of causal processes (P5), but the agent's account of their reasons (P4). While the first version of Nate's explanation is most likely insufficient to prevent thick blame (accountability), version two allows initial conclusions to be drawn about the sufficient moral quality of the explanation (P6). “If I can offer no exculpatory explanation of my action, or if the explanation I offer is inadequate, you may quite reasonably blame me […]” (Duff 2009, 980). Regarding the explanation, Duff speaks of *adequacy*, and others describe it as *sufficiency* (Tiffany 2024, 631), or *appropriateness* (Smith 2015, 113), whereby this question is addressed in this article by using (1) comprehensibility and (2) moral standards.

The objectivity of the subjective explanation is not exclusively dependent on moral standards being met or not. In the example just described, Nate could not achieve the required moral quality of the explanation if one assumes that a moral standard was violated. The harm done to Tom is difficult to reconcile with standards of carefulness (Tiffany 2024, 632) or nonmaleficence (Beauchamp and Childress 2019, 155–156), even if Nate had no intention of doing so. In many cases where a response is justifiably demanded from an actor (P1), a standard will have been violated. Nevertheless, it can be assumed that the second version of Nate's explanation will be sufficient for many people to refrain from thick blame (accountability) (Duff 2009, 980–981). But this also means that even if moral standards have been violated,^8^ an explanation can still be of sufficient moral quality. This allows the fulfillment of P6 to be recognized, and the subject is considered (P4) without simultaneously and exclusively inferring moral responsibility from causal relationships (P5) (Kropf 2026a, 4). Taken together, (1) comprehensibility and (2) moral standards function as jointly necessary, but not individually sufficient, conditions for the moral quality of an explanation. These criteria (for P6) are not intended as strict decision rules, but as evaluative guidelines that structure how explanations are assessed within practices of responsibility.

A further clarification concerns the role of intention. One could also put it this way: “[…] one is answerable for an account of one's reasons for those things that can be understood as one's settling for oneself some question(s) or as the intended or foreseen consequence of executing some intention” (Hieronymi 2008, 362). *First*, her reasoning can be understood to mean that a person's specific intention or purpose is decisive in determining whether they are answerable. However, this is problematic. Let us imagine a robot (Pepper) bumping into a nurse (Ben). Even if it seems unreasonable to ask Ben for an explanation and not Pepper, we can take this scenario further: If Ben bumps into a patient (Eva) because of Pepper's bump, and Eva spills hot coffee and injures herself, it seems conceivable that Eva would ask Ben for an explanation. However, Ben did not bump into Eva intentionally, but because of Pepper's bump—so intention can be ruled out. Let us also assume that Eva did not see the robot. Then it still seems justified (P2 or P3) that Eva can demand an explanation. If one emphasizes intention as a prerequisite for answerability, then Ben would not be answerable because there is no intention. This seems implausible to me because such a view either (a) presupposes that an answer has already been given in order to determine intentional action, and that one is then not answerable due to a lack of intention. Or (b) answerability would require that intentional action has already been identified before an explanation is demanded. Both options are implausible: either answerability is denied despite relying on the agent's explanation, or it requires prior knowledge that only the explanation itself can provide. This example suggests that answerability does not require prior intentional action, but rather the capacity to give an account of one's conduct—even where such reasons are limited or absent.

*Second*, Hieronymi's considerations should, in my opinion, be better understood as meaning that one is not answerable (only) for intentional or deliberate actions, but rather for one's own intention, attitude, and, accordingly, the reasons why one acted in a particular way (Hieronymi 2008, 366). Ben would therefore (even without the intention to push Eva) be answerable (P1) and could justify himself as follows: “I did not want to push Eva; without the robot pushing me, this would not have happened.” This results in a subjective explanation (P4) based on P2, which corresponds to a positive moral quality (P6) and goes beyond the causal connection (P5). This account is comparable—though not identical—to views that link answerability to an actor's deeper commitments: “An agent's acts and attitudes are attributable to that agent if they are appropriately related to the agent's deep commitments” (Mason 2015, 3047–3048). The pursuit of moral standards expresses what defines an actor and what reasons, principles, or moral commitments determine their actions. Put differently: “When a *person* acts, the desire by which he is moved is either the will he wants or a will he wants to be without” (Frankfurt 1971, 14). Ben's actions do not correspond to his deep commitments or his will, but this does not mean that answerability cannot be justified. And comprehensibility implies that these reasons (if there were any) are conveyed sincerely in the course of an explanation. This makes it clear why, even in such a scenario, Eva's demand for an explanation is justified, yet accountability (thick blame) for Ben is not. An explanation that is considered qualitatively appropriate, which, as described above, integrates (1) comprehensibility and (2) moral standards, could prevent (thick) blame by Eva (P7). However, it does not have to. It is also conceivable that even with a morally positive explanation, such as the one given by Ben, Eva would not refrain from taking further steps in moral terms (e.g., blame, sanctions, and disapproval) (Caruso and Morris 2017, 842). This suggests that P7 may be fulfilled independently of P6, although a positive moral quality (P6) is a strong indicator that it will be. In short, certain reactions are to be expected based on the answer, which emphasizes the relevance of giving and allowing answers.

## 3 AI‐Based Care Robots and Answerability

So far, it has been emphasized that answerability (1) is subjective and yet (2) should satisfy an objective standard. The following sections therefore revisit the central premises of the framework (especially P1, P4, and P6) and examine whether and in what sense they can be satisfied by AI‐based care robots. So, what does this mean for care robots? To answer this question, reference is made to the analytical framework, plausible objections are addressed, and the current state of care robots is considered. In simple terms, a care robot can be understood as an artificial actor that, thanks to its technical capacities and physical presence, is able to consider the needs and preferences of people requiring care (Chita‐Tegmark and Scheutz 2021, 205–206; Yew 2021, 629–630)—in a limited form. Robots enable visual or auditory communication, monitor health data, bring objects, accompany people, and allow human–robot interaction. These machines are mostly developed for vulnerable groups (e.g., elderly people, children, and people with dementia) and are increasingly performing a wide range of tasks (C. Papadopoulos et al. 2022, 251–252; Soljacic et al. 2024, 691–692). The relevance of care robots for answerability can be motivated in three respects. *First*, socially assistive robots (SARs^9^) are capable of performing a social function and specific tasks that humans (and less advanced machines) cannot perform to the same extent. Given the pace of development, a similar trend seems realistic for the future. *Second*, their use raises the normative question under which conditions their actions should be subject to demands for explanation. *Third*, although there are some proposals (Coeckelbergh 2020; Gogoshin 2021; Himmelreich 2019; Nyholm 2018) on different forms of responsibility regarding AI‐based actors, *care robot answerability* remains underresearched.^10^ The following analysis argues that care robots perform socially relevant actions, occupy roles that are not merely instrumental, and possess capacities that justify treating them as answerable—albeit with important limitations.

### 3.1 Care Robots as Answerable Actors

The first question concerns P1 and the status of care robots as potential addressees of demands for explanation. The application of this framework to care robots depends on how the actor (A) in P1 is understood. If A is restricted to human persons,^11^ care robots are excluded by definition. However, this restriction is not required by the framework itself. What matters for P1 is not personhood as such, but whether an entity can meaningfully be addressed with a demand for explanation. Care robots plausibly qualify as actors in this sense, since their actions (x) and the resulting outcomes (o) are inconceivable without their contribution (Coeckelbergh 2014, 64–65; Gogoshin 2021, 8–9; Gunkel 2020, 313–314). They therefore fall under A in P1—albeit with important restrictions. Answerability is not accountability—and explaining x is different from claiming accountability (thick blame) for x. In short, I assume that care robots can be appropriate targets of demands for explanation, even if traditional forms^12^ of accountability grounded in human moral agency do not straightforwardly apply to such systems. To illustrate this, let us take the previous example with Nate, Carry, and Tom. Carry continues to be involved as the person receiving care in the constellation with a caregiver and Tom, but Nate is now replaced by a care robot (NAO).

Let us imagine that NAO, Carry, and Tom are participating in a discussion, and NAO is moderating—which is actually already possible (Chita‐Tegmark and Scheutz 2021, 209; Rabbitt et al. 2015, 40–41). Many of the people present are speaking up. Carry tells a few unusual and seemingly funny stories about Tom, without realizing that this makes Tom feel exposed and uncomfortable. At the end of the evening, after the discussion has ended, Tom is angry with Carry and feels betrayed because experiences that were deliberately confided in him were shared with others. In short, it seems justified to claim that Tom can demand an explanation from Carry (P1) without going into further detail here—reasons (P2 and P3) follow from the premises. But how does the situation change if one assumes that NAO acts in much the same way as Carry the following day? If NAO discloses Tom's personal information in a similar way, Tom's interests are affected in the same manner (P2). The fact that NAO is not a person does not change the normative relevance of the outcome. Moreover, as argued above, answerability does not depend on intentional action. The crucial question is therefore not whether NAO intended the outcome, but whether it is appropriate to demand an explanation from NAO. In short, even in the case of a robot, P2 is fulfilled because Tom's interests are affected by the machine's actions. Denying this would require a principled reason why demands for explanation should be limited to human agents. However, neither the reference to affected interests (P2) nor to good reasons (P3) provides such a restriction. Apart from Tom's interests, NAO fulfills a specific role in the nursing home, which involves tasks and interactions with people. These interactions and the associated (technical) trust^13^ suggest that other people can also demand an explanation from NAO—apart from the general misconduct of the machine, which is another good reason.

One could argue at this point that there are no good reasons (P3) for demanding explanations from AI‐based systems because robots cannot provide explanations in the human sense. One refers, so to speak, to A in P1 and doubts that robots as actors fulfill the requirements for A. All reasons cited after that (for P1) are meaningless because of *not A*. Even if A can be equated to some extent with an intelligible target (Smith 2015, 104), a deep self (Mason 2015, 3048–3049), an agent (Jeppsson 2016, 684), or a rational being (Hieronymi 2008, 369–370), it is equally possible to describe an actor A as answerable, “[…] when it makes sense to ask why she did what she did […]” (Jeppsson 2016, 684). This functional criterion is sufficient for present purposes. Accordingly, the relevant question is not whether care robots can provide explanations in the same way as humans, but whether their responses can play a comparable role within practices of answerability. Even if such responses are technically generated and limited, they can still function as subjective explanations in the sense of P4, insofar as they provide an account of the system's action‐guiding processes. In addition, it should be noted that answerability is not about how exactly x is related to o (P5). If the subjective statement is not exclusively associated with human subjects, but rather refers to the subjectivity of the explanation, then no decisive limitation of robots can be identified here either. NAO could say, “I'm sorry Tom,” or display “I didn't mean to make you angry.” Although such a statement does not express genuine remorse, it can nonetheless serve as an answer to a justified demand for explanation.

### 3.2 Limits of Answerability: Reasons and Agency

The second issue concerns P4 and the status of reasons in AI systems. While human agents (can in principle) act on the basis of reasons in a robust sense, it is unclear whether and in what sense this applies to AI systems. While Carry may act on the basis of beliefs, intentions, or evaluative judgments, comparable reason‐based explanations cannot straightforwardly be attributed to NAO—at least not in the standard sense. Following Smith's understanding, the action of a robot does not reflect a judgment about reasons (Smith 2012, 579). However, this does not imply that robot behavior is arbitrary. On the contrary, care robots act on the basis of programmed and learned decision structures that guide action selection and allow for a limited form of flexibility (Matthias 2004, 182–183). These decision structures can be understood as functional equivalents of reasons: they guide action, differentiate between alternatives, and can be made accessible in the form of explanations, even if they do not involve reflective judgment in the human sense. If one does not merely emphasize the ontological difference between humans and machines but instead takes a functionalist approach to reasons and justifications, it becomes more plausible that advanced AI systems act on the basis of (technically understood) reasons. Smith also lowers the requirements: “[…] I mean *only* that such a creature must be able to understand the ways in which its activities might affect the ‘needs, interests, welfare, and claims of others,’ and that it must be able to make judgments about whether this *matters* in determining what to do” (Smith 2021, 342). If one regards *understanding* and *making judgments* in a minimal, non‐anthropocentric sense, aspects of these capacities can plausibly be realized—albeit in a limited and technically mediated form—by current AI systems. Put differently: “[…] the functional equivalents of intentional attitudes central to character, judgment, and quality of will in robots and AI agents can provide the basis for […] answerability […]” (Sars 2022, 3). However, it is important to note that he is not referring to the current state of the art, but to robots and AI systems that are *sufficiently autonomous* and will be (possibly) considered appropriate participants in the practice of moral responsibility *in the future*.

In practice, care robots select between alternative actions (e.g., x1 rather than x2) on the basis of programmed priorities and learned patterns (Gunkel 2020; Matthias 2004; Müller 2020). These selections are not random but structured and can therefore be reconstructed as action‐guiding considerations. In this sense, they can function as technically mediated explanations^14^ (P4). A categorical exclusion of robots because they are not persons (A in P1) or because their explanation does not represent human reasons (P4) seems wrong based on what has been said so far. This is particularly true if one separates answerability from accountability and also the necessary prerequisites for them. Champagne and Tonkens, for example, deny answerability, particularly because robots cannot feel pain and therefore punishment makes no sense (Champagne and Tonkens 2023, 2). Even though they refer explicitly to answerability, in my opinion their considerations correspond to accountability—or a mixture of the two forms. And therein lies the problem: denying accountability for machines does not imply that answerability is also irrelevant (Miller 2014, 476). Conversely, demanding a robot answer based on reasons does not mean that these machines should be held to account (thick blame) for what they did. Furthermore, like Sars, they refer to autonomous robots and not to the current state of the art—which should be considered regarding the position represented here. At present, care robots cannot be held accountable (in human terms): they do not experience remorse, cannot meaningfully accept sanctions, and lack the capacities (often) associated with moral agency. However, this does not undermine their role within practices of answerability.^15^ “[…] what someone is responsible for has significance for others who interact with or respond to them; it has significance to such responders as *this agent's* action, we may say, and may therefore license moral responses to this agent” (Edlich 2025, 21). Even if he does not refer to machines, the following conclusion can be drawn. Care robots perform certain actions based on their interaction with people receiving care, which are attributable to them and are significant. This significance does not exist for the robots themselves, but for the people involved, whose interests and needs are affected. The human–robot interaction already mentioned can lead to a relationship in which people in need of care become more active, gain motivation, or generally improve their well‐being. In this sense, their behavior can appropriately be met with demands for explanation, even if these explanations remain technically mediated^16^ and limited.

### 3.3 Moral Quality and Its Limits in Care Robots

The final issue concerns P6 and the moral quality of robot explanations—in the sense described. The moral quality of a care robot's explanation (P6) can be assessed in terms of two requirements introduced above: (1) sincerity (as a condition of comprehensibility) and (2) orientation toward moral standards. *First*, the easier question: even if certain compromises are necessary, it can be argued that robots pursue (2) moral standards.^17^ Robot behavior is structured by programmed priorities and constraints that differentiate between acceptable and unacceptable actions (Wallach and Allen 2009, 79). Without such normative structuring, systems like NAO would not distinguish between administering appropriate medication (x1) and causing harm (x2). This would not only be ethically unacceptable but also inconsistent with current design practices. Regarding the moral quality (P6) of a care robot's explanation, there is therefore no (decisive) limitation in terms of compliance with moral standards. Even if one objects that the standards described here are not those that NAO pursues based on its own *convictions*—which is debatable—but rather correspond to the specifications of the programmers, designers, and others involved in the development process, they nonetheless function as the normative reference points guiding NAO's behavior. Shifting the demand for explanation exclusively to human designers would therefore neglect the robot's situated contribution (P4) and risk reducing answerability to a purely causal analysis (P5). Furthermore, it should be noted that NAO's subjective moral standards will (or at least should) not pose any problems in terms of objective requirements, as these machines are equipped with certain guidelines provided *by humans*. And the technical flexibility emphasized above should not be understood to mean that NAO can currently develop and then follow its own and possibly arbitrary moral standards.

The more demanding question concerns (1) comprehensibility. It has been emphasized that this is not (only) about understanding an explanation, but about sincerity. NAO would therefore have to be honest, make statements that correspond to the (subjective) truth, and thus give a sincere explanation as to why Tom's personal details were disclosed. On the one hand, one might argue that care robots can satisfy a minimal notion of sincerity, insofar as their outputs reliably reflect their internal processes and are not shaped by deceptive^18^ intentions. A conscious deception regarding an answer can be ruled out due to ethical considerations and technical limitations. On the other hand, it may be argued that sincerity presupposes capacities—such as self‐reflection, intention, or moral commitment—that current AI systems lack. Hakli and Mäkelä argue that a robot's lack of history fundamentally argues against attributing responsibility to it (Hakli and Mäkelä 2019). Even if, as I assume at this point, they are referring to accountability and not answerability, it seems reasonable to conclude that their position would also deny robot answerability. But why? *First*, they assume that robots are practically incapable of acting in any other way than that specified by their programming (Hakli and Mäkelä 2019, 268–269). That this is not the case has been emphasized so far and can be substantiated by referring to their actions, which are beyond complete control (Gunkel 2020, 312). In short, they can act differently, but not arbitrarily. Second, even if one follows manipulation‐based concerns (Mele 2008, 2013), it is not clear that they straightforwardly apply to care robots. Unlike manipulated human agents, robots do not possess a prior history that is overridden. In this respect, they are more comparable to Mele's “minutelings,” whose existence is entirely shaped by external conditions, yet for whom certain forms of responsibility remain meaningful (Mele 2013, 164–165). This suggests that programmability alone does not exclude answerability, even if it limits the moral quality of explanations. Additionally, this manipulation (programming) is primarily relevant for accountability, but not necessarily for answerability—although it affects the moral quality of the answer.

What follows is that care robots may satisfy a minimal, functional notion of sincerity (P6)—namely, the reliable expression of their action‐guiding processes—but not a stronger notion that presupposes reflective endorsement or moral commitment. Beauchamp and Childress's considerations are helpful because they also focus on healthcare, and their standards are comparatively low—understanding sincerity as a basic attitude or virtue would be even more demanding (Aristoteles 2019, 40 NE 1105a–1105b). “Veracity in health care refers both to timely, accurate, objective, and comprehensive transmission of information and to the way the professional fosters the patient's or subject's understanding” (Beauchamp and Childress 2019, 328). Care robots are answerable in a minimal form, but quick answers, appropriate explanations, guidance, or even objectively comprehensible explanations are too demanding for AI (Tigard 2021, 11). Thus, it seems reasonable to conclude that the answerability of care robots has its limits. While an analysis of the respective premises did not argue against robots being able to provide answers in a technical sense, the requirement for sincerity (P6) cannot currently be met. However, there are good reasons to consider answerability meaningful in relation to care robots. Even if the moral quality (P6) of an explanation by NAO is to be assessed differently than if Carry were to answer for the same incident in a similar manner, it remains an explanation by NAO (P4). The moral quality of this explanation may ultimately lead Tom to refrain (P7) from thick blame (accountability). However, it is not only the moral quality of NAO's explanation that is decisive, because even a morally high‐quality explanation may not be sufficient to prevent Tom from blaming the machine. The assessment of accountability (thick blame)—implicitly conveyed by P6 and P7—is a different and separate issue that cannot be fully and adequately addressed here. Suffice it to say that, according to the above, it is crucial that answerability occupies a middle position between attributability and accountability. The importance of considering the subjective explanation for the attribution of thick blame (accountability) has become clear. In other words: “[…] being answerable for a norm‐violating attitude or evaluative judgment is not sufficient for being blameworthy for it” (Miller 2014, 476). Care robots can therefore be considered answerable in a limited sense: while they can provide explanations (P4) and act in accordance with embedded moral standards, they do not fully meet the requirements of sincerity associated with the moral quality of explanations (P6). These considerations suggest that the two criteria associated with P6—sincerity and orientation toward moral standards—are jointly relevant but not independently sufficient. While compliance with moral standards can be realized in current systems, sincerity can only be approximated in a minimal sense.

## 4 Conclusion

This article has examined answerability in relation to AI‐based care robots by developing and applying an analytical framework that integrates both a subjective dimension (P4) and an objective requirement (P6). Building on, but also extending, existing accounts, the framework clarifies the conditions under which demands for explanation arise and how their moral relevance can be assessed in practice. The analysis has shown that care robots can be understood as answerable in a limited and functionally specific sense. Due to their role in care settings, their interactions with vulnerable people, and the significance of their actions for affected individuals, it is both meaningful and, in many cases, justified to demand explanations from these systems. In this respect, answerability does not depend on personhood or full moral agency, but on the role an entity plays within practices of moral responsibility. At the same time, important limitations remain. While care robots can provide technically mediated explanations that reflect their action‐guiding processes (P4), they do not meet the stronger requirements associated with the moral quality of explanations (P6). In particular, sincerity can only be approximated in a minimal, functional sense, as current systems lack the capacities for reflective endorsement and genuine moral commitment. This limitation marks a decisive boundary: care robots may be answerable, but they are not accountable in the sense of being appropriate targets of blame or sanction. These findings support the view that answerability occupies an intermediate position between attributability and accountability. Recognizing this intermediate status allows for a more nuanced understanding of responsibility in human–robot interaction without conflating different forms of moral assessment. Looking ahead, it remains an open question whether more advanced AI systems—equipped with greater autonomy, learning capacities, and forms of *history*—could satisfy more demanding conditions of answerability or even approximate aspects of accountability. However, such a development is neither conceptually straightforward nor normatively unproblematic. It is important to emphasize that the value of answerability does not depend on the prospect of full moral responsibility. Rather, its relevance already lies in its contribution to transparency, trust, and the structuring of interactions in ethically sensitive care contexts. Given the vulnerability of those involved and the growing role of AI‐based systems in care practices, there are strong reasons to continue refining and critically examining the concept of answerability in this domain.

## Author Contributions

The author has accepted responsibility for the entire content of this manuscript and approved its submission.

## Funding

The author has nothing to report.

## Ethics Statement

The author has nothing to report.

## Conflicts of Interest

The author declares no conflicts of interest.

## Data Availability

Data sharing not applicable to this article as no datasets were generated or analyzed during the current study.

## Footnotes

1 Broadly speaking, approaches to moral responsibility can be divided into response‐dependent and response‐independent accounts. The former emphasize the role of reactive attitudes and social practices, whereas the latter focus on the capacities and conditions that ground responsibility. The present account is best understood as a hybrid position combining elements of both. See for example: Edlich (2025, 23–24) and Sars (2022, 4–5).

2 I draw on Zimmerman's distinction between thin and thick blame, which roughly correspond to attributability and accountability (as discussed by Watson and Shoemaker). While the focus here is on answerability, this intermediate form presupposes attributability and is necessary for the appropriate assessment of accountability, since it concerns the agent's reasons and explanations (Zimmerman 2022, 48–50; Watson 1996; Shoemaker 2011).

3 Accounts of moral responsibility typically refer to conditions such as control and knowledge (following Aristotle), often supplemented by intention and causality. These conditions are not all‐or‐nothing but admit of degrees, which is particularly relevant for assessing responsibility in the context of AI‐based systems. See for example: Mason (2015), Hieronymi (2008), Caruso and Morris (2017), Aristoteles (2019), and Malle et al. (2014).

4 P1 should be read as a minimal, practice‐dependent condition rather than a strict criterion.

5 “Action” is understood here in a broad sense, including omissions as well as attitudes or attributes insofar as they are expressed in behavior and thus become relevant for demands for explanation. Answerability concerns what is manifested in action and its outcomes, rather than unexpressed inner states. For the purposes of this article, however, the focus remains on actions, given their particular relevance in the context of AI‐based care robots. See also: Hieronymi (2008), Smith (2012), Sars (2022), and Ramsoomair (2021, 361).

6 Providing assistance to persons in danger appears to be considered a duty if (1) the person to be rescued is actually in danger, if (2) one is able to help, and if (3), on the one hand, the person to be rescued is very likely to be saved by the assistance, and (4) on the other hand, the rescuer is not highly likely to be placed in danger by the rescue. In short, the benefits outweigh the risks. See for example: Beauchamp and Childress (2019, 222–223).

7 Put simply, his reasoning can be understood as follows: the generalization of subjective standards provides insight into whether this standard is morally justifiable. “Act only according to that maxim by which you can at the same time will that it should become a universal law” (Kant 2015, GMS 421, 7–8).

8 Smith (2012), Tiffany, and Duff sometimes refer to this violation of standards or expectations when describing answerability. However, Smith (2021), Sars, and Edlich also emphasize that reactive attitudes can exist without the violation of norms. And reactive attitudes, as described above, can be seen as a reason (P3) to justify answerability. I assume that answerability can also be justified for positive actions, even though my focus is also on bad outcomes. However, this is mostly because such cases are more persuasive and also because blame and praise (or reactions to negative and positive outcomes) are not identical. See for more information: Smith (2012; 2021, 337), Edlich (2025, 21), Sars (2022), Duff (2009), Tiffany (2024), and Kropf (2026b).

9 I focus on social assistance robots (SARs), that is, embodied systems capable of interacting with users and supporting care‐related practices. Examples include Pepper, Kompai, NAO, PR2, Lio, and Care‐O‐bot. Unlike simpler devices such as the robotic seal Paro, these systems enable more complex forms of human–robot interaction and rely on advanced AI. See for example: Papadopoulos et al. (2023), Zafrani et al. (2023), Robaczewski et al. (2021), and Kropf et al. (2026).

10 There is an emerging literature on answerability and AI‐based systems, as well as broader accounts of responsibility in human–robot interaction. However, these contributions either focus on technological answerability in functional or epistemic terms, assign answerability to human agents, or develop the concept independently of care contexts. A systematic account of answerability specifically for care robots—grounded in moral practice and distinguishing it from accountability—remains underexplored. See for comparison: Champagne and Tonkens (2023), Tigard (2021), Kiener (2024), and Chen et al. (2025).

11 Most accounts of answerability implicitly or explicitly restrict its scope to persons. Smith, for example, emphasizes that explanations presuppose a competent adult agent, while others similarly focus on agents whose evaluative judgments are not determined by external manipulation. This person‐centered assumption provides an important point of departure for the present analysis (Miller 2014; Tiffany 2022, 2024; Smith 2015).

12 Whether alternative forms of accountability may become appropriate for sufficiently advanced AI systems is a separate question that goes beyond the scope of the present analysis.

13 Vallor et al. emphasize that answerability plays a key role in fostering trust in human–AI interaction, as it supports responsiveness, relational accountability, and the intelligibility of AI behavior. Similar considerations can be found in the broader literature on human–machine interaction, even if such requirements cannot be fully realized by current systems (Vallor et al. 2025; Yew 2021; Chita‐Tegmark and Scheutz 2021; Kropf et al. 2026).

14 This limitation connects to ongoing debates in explainable AI (XAI), where explanations are often characterized as approximate, or only partially faithful to underlying decision processes. Rather than requiring full transparency, the present account of answerability adopts a more practice‐oriented perspective: what matters is whether such explanations can function as meaningful responses within contexts of moral evaluation and social interaction. See for example: Hulsen (2023) and Stange et al. (2022).

15 Current AI systems already implement basic forms of answerability, for instance through natural language processing (NLP) models that generate context‐sensitive responses and domain‐specific explanations (e.g., in healthcare settings). While such answers remain limited and cannot be equated with human explanations, they demonstrate that answerability is already realized in a minimal, technically mediated form, and may be further developed in more advanced systems (Gupta et al. 2022; Heindrich et al. 2025; Law et al. 2019; Yuan et al. 2022).

16 A related concern is that many AI‐generated explanations are post hoc and may not faithfully represent the underlying causal processes. However, this limitation does not undermine the present account. Answerability (P4) does not aim at a full reconstruction of the causal relation between action and outcome (P5), but at providing a contextually meaningful account of why an action occurred. In this sense, even post hoc explanations can be sufficient for answerability, as long as they enable agents to respond to justified demands for explanation within social and moral practices. Moreover, this limitation is not unique to artificial agents: human explanations are likewise often reconstructive and shaped by retrospective interpretation. See also: Davidson (1963) and Smith (2012).

17 A common approach is to implement moral guidelines in AI systems through decision‐making architectures or algorithms (e.g., Sorell and Draper; Allen and Wallach; and van Wynsberghe). Critics argue, however, that this does not suffice for moral agency, since key moral capacities cannot be technologically realized (e.g., Hakli and Mäkelä; Champagne and Tonkens). This distinction is important for the present argument, which does not require full moral agency (Hakli and Mäkelä 2019; Sorell and Draper 2014; Wallach and Allen 2009; Van Wynsberghe 2013; Champagne and Tonkens 2023).

18 While some recent work suggests that advanced AI systems may exhibit behavior that resembles strategic misrepresentation or “deceptive” outputs, this does not imply intentional deception in a strong sense, but rather reflects optimization processes that remain compatible with the limited notion of sincerity defended here (Hagendorff 2024; Ranisch and Haltaufderheide 2025).
